# Speckle pattern analysis with deep learning for low-cost stroke detection: a phantom-based feasibility study

**DOI:** 10.1117/1.JBO.30.5.056003

**Published:** 2025-05-07

**Authors:** Avraham Yosovich, Sergey Agdarov, Yafim Beiderman, Yevgeny Beiderman, Zeev Zalevsky

**Affiliations:** a Bar-Ilan University Faculty of Engineering and the Nanotechnology Center, Ramat-Gan, Israel; b Holon Institute of Technology, Faculty of Electrical and Electronics Engineering, Holon, Israel

**Keywords:** stroke, photonics, deep learning, remote sensing, speckle pattern

## Abstract

**Significance:**

Stroke is a leading cause of disability worldwide, necessitating rapid and accurate diagnosis to limit irreversible brain damage. However, many advanced imaging modalities (computerized tomography, magnetic resonance imaging) remain inaccessible in remote or resource-constrained settings due to high costs and logistical barriers.

**Aim:**

We aim to evaluate the feasibility of a laser speckle–based technique, coupled with deep learning, for detecting simulated stroke conditions in a tissue phantom. We investigate whether speckle patterns can be leveraged to differentiate healthy from restricted flow states in arteries of varying diameters and depths.

**Approach:**

Artificial arteries (3 to 6 mm diameters) were embedded at different depths (0 to 10 mm) within a skin-covered chicken tissue, to mimic blood-flow scenarios ranging from no flow (full occlusion) to high flow. A high-speed camera captured the secondary speckle patterns generated by laser illumination. These video sequences were fed into a three-dimensional convolutional neural network (X3D_M) to classify four distinct flow conditions.

**Results:**

The proposed method showed high classification accuracy, reaching 95% to 100% for larger vessels near the surface. Even for smaller or deeper arteries, detection remained robust (>80% in most conditions). The performance suggests that spatiotemporal features of speckle patterns can reliably distinguish varying blood-flow states.

**Conclusions:**

Although tested on a tissue phantom, these findings highlight the potential of combining speckle imaging with deep learning for accessible, rapid stroke detection. Our next steps involve direct *in vivo* experiments targeting cerebral arteries, acknowledging that additional factors such as the skull’s optical properties and the likely need for near-infrared illumination must be addressed before achieving true intracranial applicability. We also note that examining the carotid artery *in vivo* remains a valuable and practical step, given its superficial location and direct relevance to stroke risk.

## 1 Introduction

Stroke is a severe medical condition that occurs when the blood supply to a part of the brain is cut off or significantly reduced, leading to the death of brain tissues due to lack of oxygen.^1^^,^^2^ Globally, stroke is a leading cause of mortality and long-term disability. Strokes can be broadly categorized into ischemic and hemorrhagic. The condition, known as a transient ischemic attack (TIA), is often regarded as a warning stroke.^3^

Ischemic stroke, which accounts for ∼87% of all strokes, occurs when a blood clot obstructs a blood vessel supplying the brain. The clot may form within a blood vessel in the brain (thrombotic stroke) or travel from another body part to the brain (embolic stroke). Atherosclerosis and the buildup of fatty deposits on blood vessel walls often contribute to clot formation.^4^

Hemorrhagic stroke occurs when a weakened brain blood vessel ruptures. There are two main types of hemorrhagic strokes: intracerebral (the most common type, occurring when an artery in the brain bursts, flooding the surrounding tissue with blood) and subarachnoid (bleeding into the subarachnoid space, which is the area between the brain and the covering tissues).^5^ A TIA occurs when a temporary block in blood flow to the brain happens. The blood clot and TIA symptoms last for a short period, unlike other types of stroke, which have more prolonged effects.^6^

Having discussed the various types of strokes, it is essential to understand the underlying hemodynamic factors that contribute to these conditions. Understanding blood flow dynamics in cerebral arteries and veins is crucial as disruptions in either system can lead to severe neurological consequences. The brain’s arterial system, which includes the internal carotid and vertebral arteries, delivers oxygenated blood to the brain. These arteries contribute to the formation of the circle of Willis, a network that ensures consistent blood flow to the brain. Blockages in arteries, often caused by atherosclerosis or embolism, can result in ischemic strokes, highlighting the importance of arterial health in stroke prevention.

On the other hand, the brain’s venous drainage system, in which veins are integrated into the dura mater to form venous sinuses, plays an equally important role in removing deoxygenated blood. Any obstruction in this system, such as cerebral venous sinus thrombosis, can lead to increased intracranial pressure and edema, resulting in stroke-like symptoms.^7^ Both the arterial and venous systems must function properly to maintain cerebral homeostasis as obstructions in either can severely impact brain function and cause long-term neurological damage.

The dynamics of blood flow can be mathematically represented using Poiseuille’s law:

$$\mathrm{BF}=\frac{\Delta P}{R},$$ (1)

where BF is the blood flow, ΔP is the pressure drop across the vessel, and R is the resistance. The brain’s average cerebral perfusion pressure is ∼60 to 80 mmHg,^8^ and any significant deviation from this range can impact brain function.

The dynamics of blood flow can be mathematically represented using Poiseuille’s law, which illustrates how minor changes in vessel diameter can profoundly affect blood flow. According to this law

$$Q=\frac{\Delta P\pi R^{4}}{8\;\eta \;L},$$ (2)

where Q is the flow rate, R is the radius of the vessel, eta is the viscosity of the blood, and L is the length of the vessel. This relationship emphasizes that even small decreases in vessel radius can significantly reduce blood flow due to the $R^{4}$ dependence, which is critical in the context of stroke where vessel obstruction or narrowing occurs

It is vital to identify the signs and symptoms of a stroke as soon as possible because early treatment can reduce brain damage and possible complications. Typical symptoms include trouble with speaking and understanding, paralysis or numbness of the face, arm, or leg, trouble seeing in one or both eyes, headache, and difficulty walking. The treatment of stroke depends on its type. Ischemic strokes can be treated with drugs that dissolve the blood clot obstructing blood flow to the brain, whereas hemorrhagic strokes require interventions to stop the bleeding. The sooner a stroke patient receives treatment, the less damage will occur; hence, the critical importance of time in stroke treatment is often encapsulated in the phrase “time is brain.”

Diagnosing a stroke promptly and accurately is critical to implementing effective treatment and minimizing potential neurological damage. Medical practitioners employ various diagnostic methods, each providing unique insights into the patient’s condition.

The diagnostic process often begins with a physical and neurological exam. These exams evaluate the patient’s overall health and the functioning of their nervous system, respectively. Listening to the heart and checking the blood pressure can give initial indications of cardiovascular health. Blood tests are also conducted to gain insights into the blood clotting speed, sugar levels, and possible infections, among other parameters.^9^^–^^11^

Next, imaging techniques such as computerized tomography (CT) scans, magnetic resonance imaging (MRI), and US Doppler imaging are utilized. A CT scan uses a series of X-rays to create a comprehensive brain image, which can reveal bleeding, ischemic stroke, tumors, and other brain conditions. In some cases, a dye may be injected into the bloodstream to enable a more detailed view of the blood vessels in the neck and brain through the procedure known as computerized tomography angiography.

MRI employs powerful radio waves and magnetic fields to generate a detailed view of the brain. It can detect brain tissue damaged by an ischemic stroke and brain hemorrhages. Like CT scans, MRI scans may involve injecting a dye into the bloodstream to highlight the arteries, veins, and blood flow through a procedure called magnetic resonance angiography or magnetic resonance venography.^12^^,^^13^

In addition, Doppler ultrasound (US) imaging is extensively employed to assess carotid blood flow in the neck, making it an important tool for stroke risk evaluation. By measuring flow velocities and detecting potential stenosis or plaque buildup in the carotid arteries, Doppler US provides real-time hemodynamic insights without exposing patients to ionizing radiation. Owing to its noninvasive nature, relatively low cost, and high portability, Doppler ultrasound is well-suited for both routine screening and early intervention in hospital and outpatient settings. Identifying significant carotid stenosis through Doppler ultrasound can facilitate timely surgical or pharmacological interventions to prevent ischemic events.^14^ However, similar to CT and MRI, Doppler ultrasound has constraints that may limit its utility as a standalone modality. It typically requires a trained operator to obtain reproducible measurements, can yield suboptimal results in patients with challenging anatomies or extensive calcification, and primarily focuses on larger, superficial vessels thus providing limited information about deeper cerebral circulation or microvascular flow. Consequently, although Doppler US is a valuable component of stroke diagnostics, these inherent limitations underscore the need for additional or complementary methods that deliver a broader perspective on cerebral hemodynamics without relying on specialized ultrasound expertise or favorable patient anatomy.

Despite their effectiveness, the mentioned methods are expensive, require very skilled personnel, and are not always accessible, particularly in remote or under-resourced areas. In addition, the time required to perform these imaging tests can delay critical treatment. Therefore, there is a pressing need for accessible, swift, and remote stroke detection methods. Research in this field is vital to developing technologies that can reliably and quickly diagnose strokes across various environments, minimizing treatment delays and improving outcomes.

A particular laser-based system^15^ has demonstrated the capacity to recognize speech signals remotely, blood pulse pressure,^16^ heartbeats,^17^ blood oxygen saturation,^18^ glucose detection,^19^ human senses,^20^ and intraocular pressure.^21^ This method analyzes the speckle patterns captured in the far field using a defocused camera to extract the tilting movement of the investigated surface by performing an optical Fourier transform. In this process, the tilting movement of the illuminated tissue is transformed into a transversal movement of the speckle patterns, which can be readily retrieved using correlation-based operations to produce a displacement graph or by applying deep learning models to the raw speckle pattern video, containing a sequence of the captured frames. It allows several biomedical data to be extracted from their dynamics. The field (amplitude and phase) distribution of the speckle pattern could be expressed as follows:^22^

$$T_{m}(x_{o},y_{o})=\iint \exp[i\phi (x,y)]\exp[\frac{\pi i}{\lambda Z_{1}}((x-x_{o}{)}^{2}+(y-y_{o}{)}^{2})]dxdy\;=A_{m}(x_{o},y_{o})\exp[i\psi (x_{o},y_{o})],$$ (3)

where (x and y) are the coordinates in the transversal plane and Z is the axial axis. *λ* represents the optical wavelength, affecting the resolution of the speckle patterns. $\phi (x_{o},y_{o})$ is the random phase due to surface roughness, determining how light interferes to form the speckle pattern. $\psi (x_{o},y_{o})$ is the phase of the speckle field, and $Z_{1}$ denotes the distance between the object and the imaging plane, impacting light scattering. $A_{m}(x_{o},y_{o})$ is the amplitude of the speckle field, controlling the intensity, whereas $T_{m}(x_{o},y_{o})\;$ represents the total field distribution, combining both amplitude and phase at each point. The intensity of the obtained speckle image being:^23^

$$I(x_{s},y_{s})=|\iint T_{m}(x_{o},y_{o})h(x_{o}-Mx_{g},y_{o}-My_{g})dx_{o}dy_{o}{|}^{2},$$ (4)

where h is the spatial impulse response, *M* is the inverse of the magnification of the imaging system, and $\left(x_{s},y_{s}\right)$ is the sensor plane coordinates’ set.

Another speckle-based approach for blood flow visualization and analysis was carried out by laser speckle contrast analysis (LASCA).

LASCA is an optical imaging technique used in biomedical research and other fields to study blood flow dynamics. LASCA relies on the interaction between laser light and moving red blood cells. When laser light interacts with moving red blood cells, the reflected speckle patterns undergo contrast variation caused by intermittent blurring. By analyzing the speckle pattern contrast, LASCA allows us to measure the blood flow. It is usually applied to the measurements in different blood vessels.^24^^–^^26^ This method has been also applied to analyze peripheral arterial disease.^27^ However, the measurement precision of this method is restricted by the arterial depth.

Other researchers have developed diverse phantom models to simulate realistic tissue conditions for stroke detection using advanced imaging techniques. For example, Ref. 28 presented a microwave‐based head phantom composed of multiple layers that accurately mimic the anatomical and dielectric properties of human tissues such as skin, bone, and brain matter, thereby offering a nuanced representation of tissue heterogeneity for microwave imaging-based stroke estimation. Similarly, Ref. 29 described a realistic three-dimensional human head phantom explicitly designed for hemorrhagic stroke detection. By incorporating detailed anatomical features that replicate the spatial and dielectric characteristics of the human head, this phantom provides an enhanced platform for testing imaging systems under conditions that closely mirror clinical scenarios.

Complementing these microwave-based approaches, Refs. 30–32 focused on optical speckle imaging combined with deep learning techniques to assess blood flow dynamics in tissue phantoms. In Ref. 30, a phantom constructed from pork fat with an embedded plastic tube simulates a blood vessel, enabling the evaluation of flow and vessel depth using a three-dimensional convolutional neural network—although pulsatile dynamics are not incorporated. By contrast, Ref. 31 employed a tissue phantom with stable optical properties that mimic the dynamic characteristics of blood flow, providing a controlled environment for assessing speckle contrast imaging and the sensitivity of deep learning algorithms in quantifying flow. Moreover, Ref. 32 introduced a tunable dynamical tissue phantom for laser speckle imaging, where key parameters such as scattering, absorption, and flow dynamics can be adjusted to closely replicate various physiological conditions. This tunability reinforces the efficacy of deep learning in reliably processing speckle images, ultimately underscoring the potential of combining optical imaging with AI for noninvasive, remote stroke diagnostics.

In this paper, we describe a phantom-based feasibility study investigating various arterial flow rates by AI speckle pattern analysis. Although the ultimate clinical goal includes intracranial stroke detection *in vivo*, the current work focuses on proof-of-concept under simplified conditions without a skull layer. We discuss how future steps will require near-infrared illumination and additional optical considerations to address deeper, more realistic brain imaging.

## 2 Materials and Methods

### 2.1 Stroke Detection System Design

The experimental setup aimed to simulate and measure blood flow velocity using secondary speckle pattern analyses. It was designed to capture speckle patterns resulting from laser illumination of a tissue phantom, enabling the detection of subtle changes indicative of different blood flow states.

The tissue phantom was a chicken tissue sample with artificial arteries embedded beneath the skin at a varied depth to mimic human blood vessels. These arteries had two internal diameters, 3 and 6 mm, with a corresponding wall thickness of 0.3 and 0.5 mm. Each artery was placed at different depths beneath the chicken skin to provide experimental conditions. For arteries with 3 mm diameter, the depths, measured from the vessel’s top, were 0, 5, and 10 mm under the skin. For arteries with a 6 mm diameter, the depths were 0 and 5 mm. Although the chosen diameters and depths reflect those of several important cerebral arteries [e.g., middle cerebral artery (MCA) or internal carotid artery (ICA)], our phantom does not include any skull-like layer. Therefore, the optical scattering and absorption introduced by bone in actual intracranial scenarios are not accounted for here.

These arteries’ diameters and depths were selected to replicate conditions under which blood flow can be compromised in stroke cases. Blood vessels in the human body vary widely in diameter, and smaller vessels are more likely to become occluded, leading to ischemic strokes. Consequently, 3 mm arteries were chosen to represent smaller vessels commonly affected by reduced blood flow. In humans, MCA (∼2 to 3 mm diameter) is often involved in ischemic events due to its large territory and direct flow from the internal carotid artery.^33^^,^^34^ Similarly, the lenticulostriate arteries (LSAs) (∼1 to 2 mm), which branch off from the MCA, are also prone to stroke.^35^

Arterial blood flow velocities likewise vary with vessel size and physiological conditions. For instance, the normal resting flow in the MCA typically ranges between 40 and 80 cm/s,^36^ in the ICA around 20 to 40 cm/s,^37^ and in the LSAs ∼7 to 12 cm/s.^28^ By contrast, 6 mm diameter arteries were selected to represent larger vessels such as the ICA (∼5 to 6 mm) and the basilar artery (∼3 to 7 mm).^38^^,^^39^ These larger arteries supply critical brain regions and, when compromised by occlusions or ruptures, can lead to more extensive ischemic or hemorrhagic strokes.

Table 1 presents these typical diameters, flow velocities, and stroke relevance. The ranges used in this study (4.2 to 111 cm/s for the 3 mm arteries and 2.7 to 78 cm/s for the 6 mm arteries) span both typical and extreme physiological conditions reported for smaller and larger cerebral vessels. This ensures that our phantom setup reflects real-world scenarios, including partial and complete occlusions that might lead to stroke. In addition, by placing these vessels at depths of 0 to 10 mm, we replicated both superficial and deeper vessel conditions a factor crucial for understanding how light scattering and absorption influence speckle patterns.

**Table 1:** Typical diameters, flow velocities, and stroke relevance for selecting cerebral arteries

Artery	Typical diameter (mm)	Typical flow velocity (cm/s)	Stroke relevance
MCA	∼2 to 3	40 to 80	Common site for ischemic strokes. Supplies large lateral portions of the brain
LSAs	∼1 to 2	7 to 12	Small penetrating branches are frequently involved in lacunar strokes
ICA	∼5 to 6	20 to 40	Large-vessel occlusions can affect anterior circulation broadly, causing extensive infarcts
Basilar artery	∼3 to 7	∼30 to 60	Supplies brainstem and cerebellum. Occlusion can be catastrophic

**Fig. 1:**
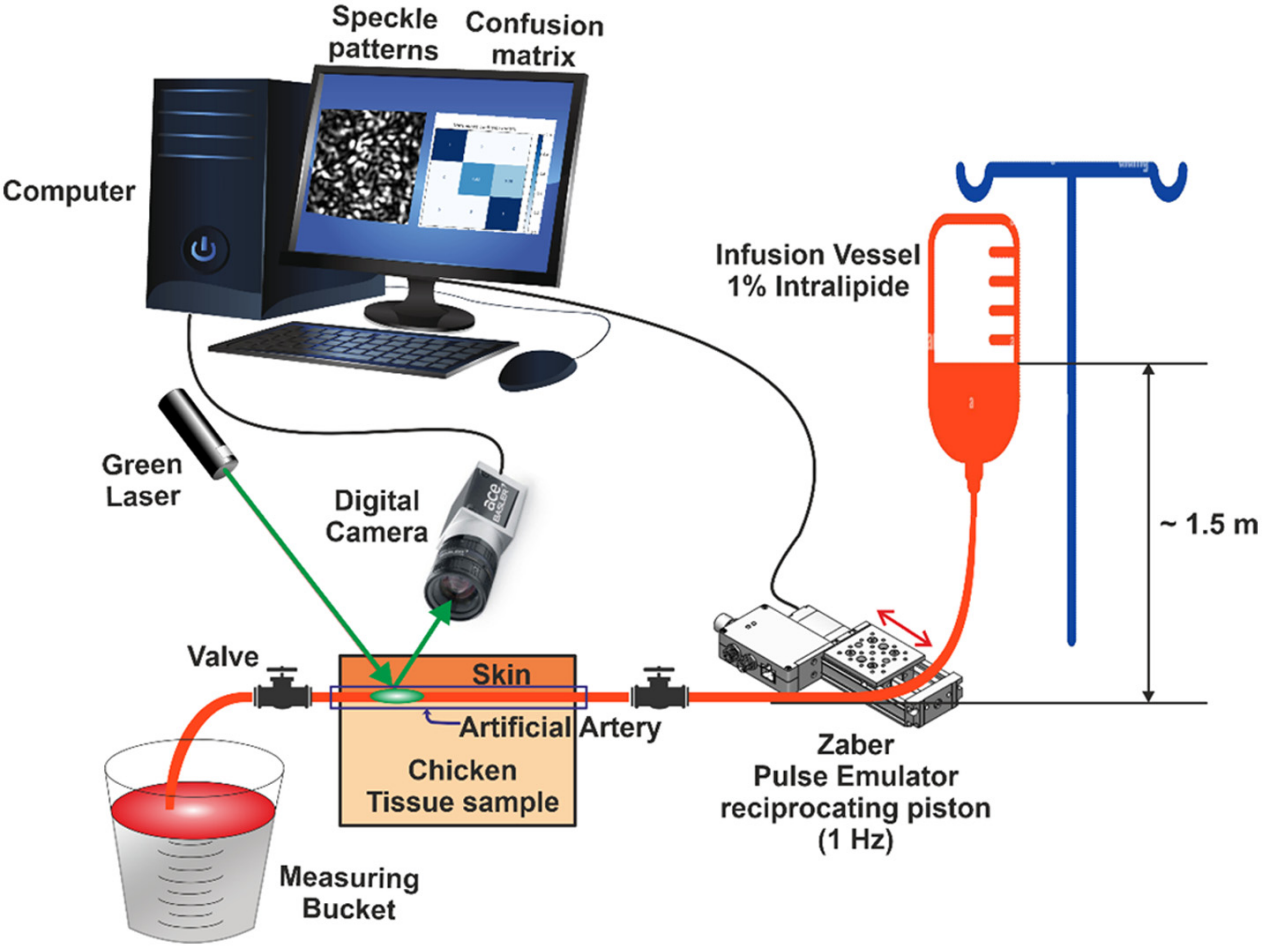
Phantom-based experimental setup schematic. A 532 nm laser illuminates embedded arteries in chicken tissue, with a defocused Basler acA1440-22um camera capturing speckle patterns. Flow is regulated by an intralipid infusion bag and pulsation by a motorized stage.

**Fig. 2:**
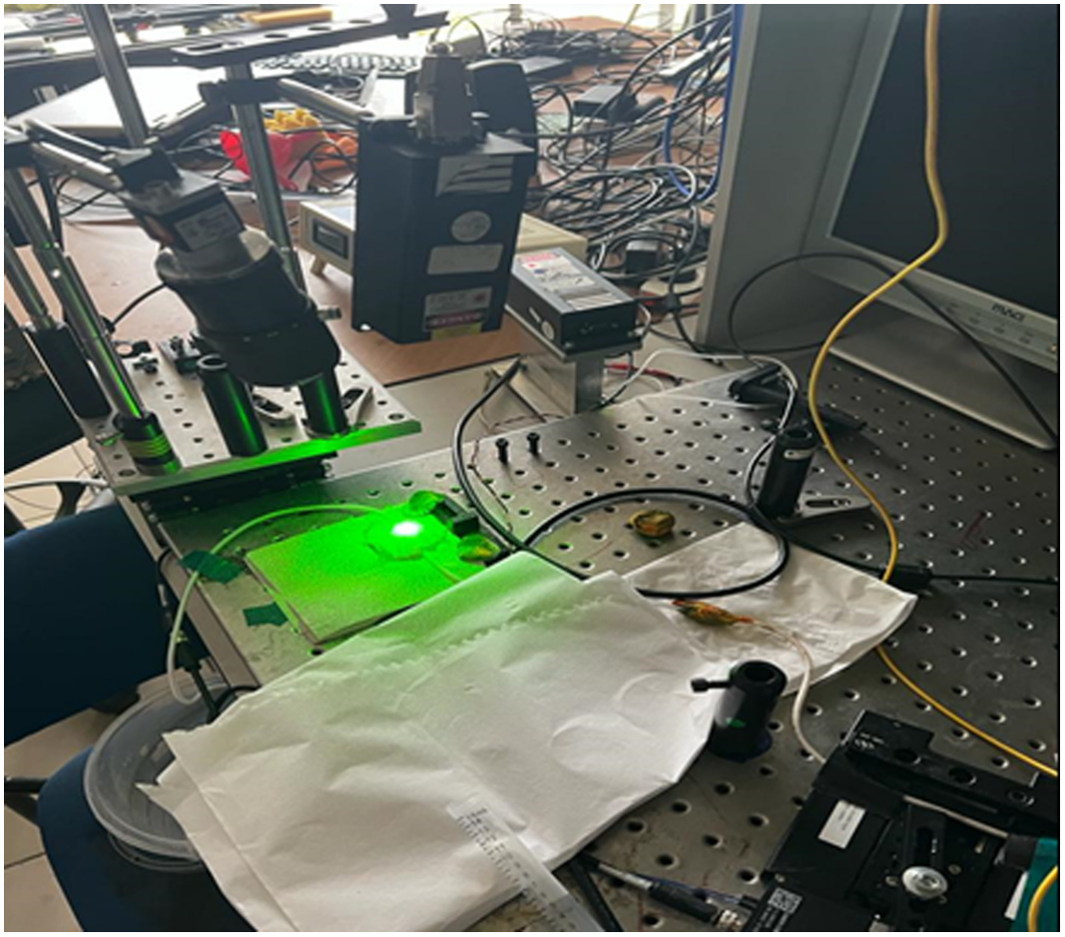
Laboratory photo of the phantom-based experimental setup. A photograph of the real arrangement. The laser illuminates the tissue from above while the defocused camera records speckle patterns. Flow and pulsation are driven by the same components shown in Fig. 1.

The setup scheme and the laboratory configuration are presented in Figs. 1 and 2.

A green laser with a wavelength of 532 nm was used as the light source. This choice is suitable for the present phantom-based validation, but near-infrared (NIR) illumination may be required for truly intracranial or deeper tissue measurements to overcome the stronger scattering and absorption that occur in the presence of bone and thicker tissue. The laser was carefully aligned to illuminate the chicken skin directly above the tested arteries. The laser illumination was crucial for generating speckle patterns on the tissue surface, which are sensitive to underlying changes in flow dynamics. The laser beam covered a small area, ∼3  mm in diameter, to ensure precise illumination and minimize extraneous reflections.

A 1% intralipid solution was contained in an infusion bag suspended 150 cm above the phantom, creating an approximate static pressure of 115 mmHg. This infusion setup remained fixed throughout all experiments. To simulate different flow rates from full occlusion (“zero flow”) to high flow, we controlled an inline valve on the infusion line:

• Zero flow (full occlusion): The valve was completely closed, halting all flow through the artificial artery.
• Restricted flow: The valve was partially closed, yielding volumetric measurements that indicated a significant drop in flow velocity compared with normal physiological ranges.
• Medium flow: The valve was opened further to achieve moderate velocities, typically around 20 to 40 cm/s (for the 6 mm arteries).
• High flow: The valve was opened nearly fully, producing velocities at or above the higher bounds of typical cerebral flow (e.g., up to ∼111  cm/s for the 3 mm arteries).

After each valve adjustment, we measured the outflow volume over a fixed time (volumetric method) and computed the corresponding velocity. These measured velocities served as ground truth labels zero, restricted, medium, or high for our deep learning models.

A motorized linear stage Zaber unit, used as a pulse emulator, contains a reciprocating piston, periodically contracting the supplying pipe to simulate the flow variation related to heartbeats at a frequency of 1 Hz. This mechanism mimicked the pulsatile nature of blood flow, which is essential for generating realistic speckle pattern variations.

A Basler digital camera (acA1440-22um) was positioned 50 cm from the chicken tissue to capture speckle patterns. The defocused camera was configured to capture images at a frame rate of 300 frames per second (FPS) with a resolution of 224×224  pixels. This high-resolution setup allowed for detailed analysis of the speckle patterns, capturing subtle variations caused by changes in the flow conditions. The camera’s high frame rate was essential for resolving the fast dynamics of the speckle pattern fluctuations.

A computer was employed to save and process the recorded video data. Custom software was used for real-time data acquisition and preliminary processing of the video files.

The experimental procedure involved several steps to ensure consistent and accurate data collection. The tissue phantom was initially prepared by embedding the artificial at a selected depth within the tissue covered by skin. The laser was then aligned to illuminate the target area precisely, and the intralipid solution was flown through the arteries to simulate varying flow rates. The pulse generator applied periodic pressure on the artery to mimic heartbeats, creating dynamic changes in the speckle patterns. Speckle pattern videos were recorded using the Basler digital camera at the specified frame rate and resolution, capturing the intricate details of the speckle fluctuations.

Speckle pattern videos were recorded for each artery diameter and depth combination, and measurements were taken to evaluate the system’s ability to detect flow velocities. The raw labels from the experiments, including video tags, video labels, velocity, and vessel depth, were recorded and analyzed. The measurements for the 3 mm arteries at depths of 0, 5, and 10 mm and the 6 mm arteries at depths of 0 and 5 mm provided a robust dataset for validating the experimental setup and the analysis techniques. The data collected provided a comprehensive dataset for developing and testing deep learning models to classify different flow states. The recorded speckle patterns were the basis for subsequent analysis, ensuring that the generated data were realistic and relevant. We simulated several blood flow conditions: full ischemic stroke with zero flow velocity, partial artery blockage with a reduced flow, and nominal flow through a healthy vessel.

This comprehensive setup allowed for the controlled simulation and measurement of blood flow characteristics, providing valuable data for subsequent analysis. The experimental design ensured that the generated data were realistic and relevant, supporting the development of robust and effective diagnostic tools for blood flow analysis.

**Fig. 3:**
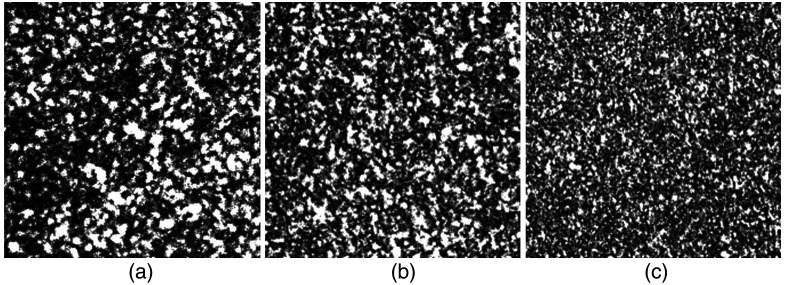
Sample speckle patterns from 3 mm arteries at 0, 5, and 10 mm depth. Representative frames at (a) 0, (b) 5, and (c) 10 mm show progressively increased scattering and reduced speckle contrast with greater depth.

Below, some examples of our recorded defocused speckle patterns are provided (Fig. 3).

### 2.2 Data Processing

All experiments were conducted in several test sessions, with each chicken tissue sample being tested in one continuous session. The experimental dataset comprised ∼906,000 frames, each contributing numerous distinct videos with varying flow conditions. Each video lasted ∼33  s and was captured at 300 FPS, resulting in 9900 frames per video. Each video frame comprised a 2D array with a spatial resolution of 224×224  pixels.

Three videos were recorded for each measurement condition: two for training and one for testing. The last video recorded for each measurement was always reserved for testing. This approach ensured that the test data were completely unseen during the training phase, providing a robust evaluation of the model’s performance.

To prepare the data for analysis, the following preprocessing steps were applied.

#### 2.2.1 Normalization

The video’s grayscale frames were normalized by dividing by 255 to bring the pixel values to a range between 0 and 1. This normalization method was chosen to ensure consistency in pixel value ranges, which helps stabilize and improve the convergence of the deep learning models during training. Normalizing the pixel values to [0, 1] makes the input data more suitable for the activation functions used in neural networks, such as ReLU, which perform better with normalized inputs.

#### 2.2.2 Chunking

The normalized frames were then segmented into chunks. Each chunk comprised 64 consecutive frames, creating a temporal window for analysis. A stride of 32 frames was used between chunks, allowing overlapping segments to enhance temporal continuity and information retention. This chunking method ensured that each segment of video data provided sufficient temporal information for the model while allowing overlap between consecutive chunks to enhance continuity and train with more data.

#### 2.2.3 Channel conversion

To prepare the data for input into the deep learning model, the grayscale frames were repeated three times to create a three-channel input. This step was necessary because most convolutional neural networks are pretrained on large datasets such as ImageNet and expect three-channel (RGB) inputs. By replicating the grayscale channel across the three input channels, we adapted our single-channel image chunks to match the input format required by these networks without losing the grayscale information.

#### 2.2.4 Dataset creation

Videos from different recording sessions were combined to form an overall dataset. We created datasets for the 3 mm diameter and 6 mm diameter arteries. During training, 10% of the training chunks were randomly selected and used for validation. This validation subset ensured that the model’s performance was monitored using unseen data during training, helping to prevent overfitting and ensuring that the model generalized well.

### 2.3 Deep Learning for Stroke Detection

The input data for the speckle pattern classification consisted of chunks of grayscale video frames, normalized, and structured, as described in Sec. 2.2. These data were used to train several models from the X3D family,^40^ specifically X3D_S, X3D_XS, and X3D_M. After comparative evaluations, we chose the X3D_M model for its balance between performance and computational efficiency, making it well suited for stroke detection.

The X3D_M model is part of the X3D family of models developed by Facebook AI (Meta). These models are designed for efficient video recognition, leveraging 3D convolutional neural networks to capture spatiotemporal features in video data.^40^^,^^41^

The X3D architecture scales spatial and temporal dimensions for flexible and efficient video data processing.

The X3D_M model is designed for efficient video recognition. It uses 3D convolutions to capture both spatial and temporal features in video data.^42^

The model architecture consists of the following layers:

Input layer: The input to the model is a chunk of video frames.

3D convolutional layer (Conv3D): This layer applies 3D convolutions to the input video frames. The first Conv3D layer has 24 filters with a kernel size of (3, 3, 3), stride (1, 1, 1), and padding (1, 1, 1) to preserve the spatial and temporal dimensions.

Batch normalization (BatchNorm3D): Following the Conv3D layer, batch normalization is applied to stabilize and accelerate the training process by normalizing the output of the convolutional layer.

ReLU activation (ReLU): A ReLU activation function is applied to introduce nonlinearity, allowing the model to learn complex patterns in the data.

Intermediate 3D convolutional blocks: Multiple 3D convolutional blocks are stacked, each consisting of a Conv3D layer, BatchNorm3D, and ReLU activation. These blocks gradually increase the number of filters and reduce the spatial dimensions through pooling layers, capturing increasingly abstract features from the input video.

Pooling layers: Pooling layers (e.g., max pooling or average pooling) are interspersed between the convolutional blocks to reduce the spatial dimensions. This allows the model to focus on the most relevant features while reducing computational complexity.

Fully connected layers: After the 3D convolutional and pooling layers series, the feature maps are flattened and passed through fully connected (dense) layers. These layers further process the extracted features to produce the final classification output.

Output layer: The final layer is a fully connected layer with a softmax activation function, providing the probability distribution over the target classes

The dataset was divided into training and testing sets, with the last video recorded for each measurement condition reserved for testing. The training process involved the following steps:

Batch size and precision: A batch size of 8 was used, and half-precision (16-bit floating point) was employed to allow for larger batch sizes and faster computations without significantly compromising model accuracy.

Epochs: The model was trained for 20 epochs, ensuring sufficient training time to learn the complex patterns in the data.

Optimizer: The Adam optimizer was used with an initial learning rate of 0.001. Adam is well suited for this task due to its adaptive learning rate capabilities, which help it converge faster and avoid local minima.

Loss function: Categorical cross-entropy was used as the loss function. This is defined as:

$$L_{\mathrm{CE}}=-\sum_{i=1}^{n}t_{i}\log(p_{i}),\;\mathrm{for}\;n\;\mathrm{classes}$$ (5)

where $t_{i}$ is the ground truth label for class *i* and $p_{i}$ is the predicted probability for class *i*.

Use of pretrained weights: Although the X3D_M model is typically used with pretrained weights on large datasets such as Kinetics-400, we trained the model from scratch for this study. This approach ensured the model learned features specific to the speckle pattern data rather than relying on features from unrelated datasets. We experimented using pretrained weights from the Kinetics-400 dataset and applied transfer learning to our speckle videos, but the results were unsatisfactory. This led us to conclude that training the model from scratch was necessary for optimal performance in our application.

This model’s architecture allows for efficient processing of the input video chunks, making it ideal for real-time stroke detection applications. The lightweight nature of the X3D_M model makes it particularly suitable for deployment in real-time diagnostic systems where computational resources may be limited.

The chosen model and training parameters ensured that the X3D_M model could accurately classify speckle pattern videos, providing a reliable tool for detecting blood flow abnormalities associated with a stroke. The structured approach to data preprocessing and model training ensured that the results were accurate and reliable, contributing valuable insights into detecting and classifying different blood flow states.

## 3 Results

This section presents the results of the speckle pattern analysis conducted using tissue phantoms with embedded artificial blood vessels of different diameters and installation depths. The aim was to evaluate the system’s ability to detect blood flow changes indicative of stroke conditions. The results are categorized based on the diameter and depth of the arteries.

### 3.1 Speckle Pattern Analysis for 3 mm Diameter Arteries

The 3 mm diameter arteries DL analysis showed varying success rates of intralipid velocity detection based on the depth at which the arteries were placed. The selected vessel installation depths under the skin were 0, 5, and 10 mm. The system’s ability to detect different flow conditions (zero flow, restricted flow, medium, and high flow) was assessed at each depth.

At a vessel depth of 0 mm under the skin, the model achieved a success rate of close to 100% for detecting zero, restricted, and medium flow. This high success rate indicates the system’s robustness in detecting an ischemic stroke. The success rate in detecting a stroke at 10 mm depth is also very high (89%).

**Fig. 4:**
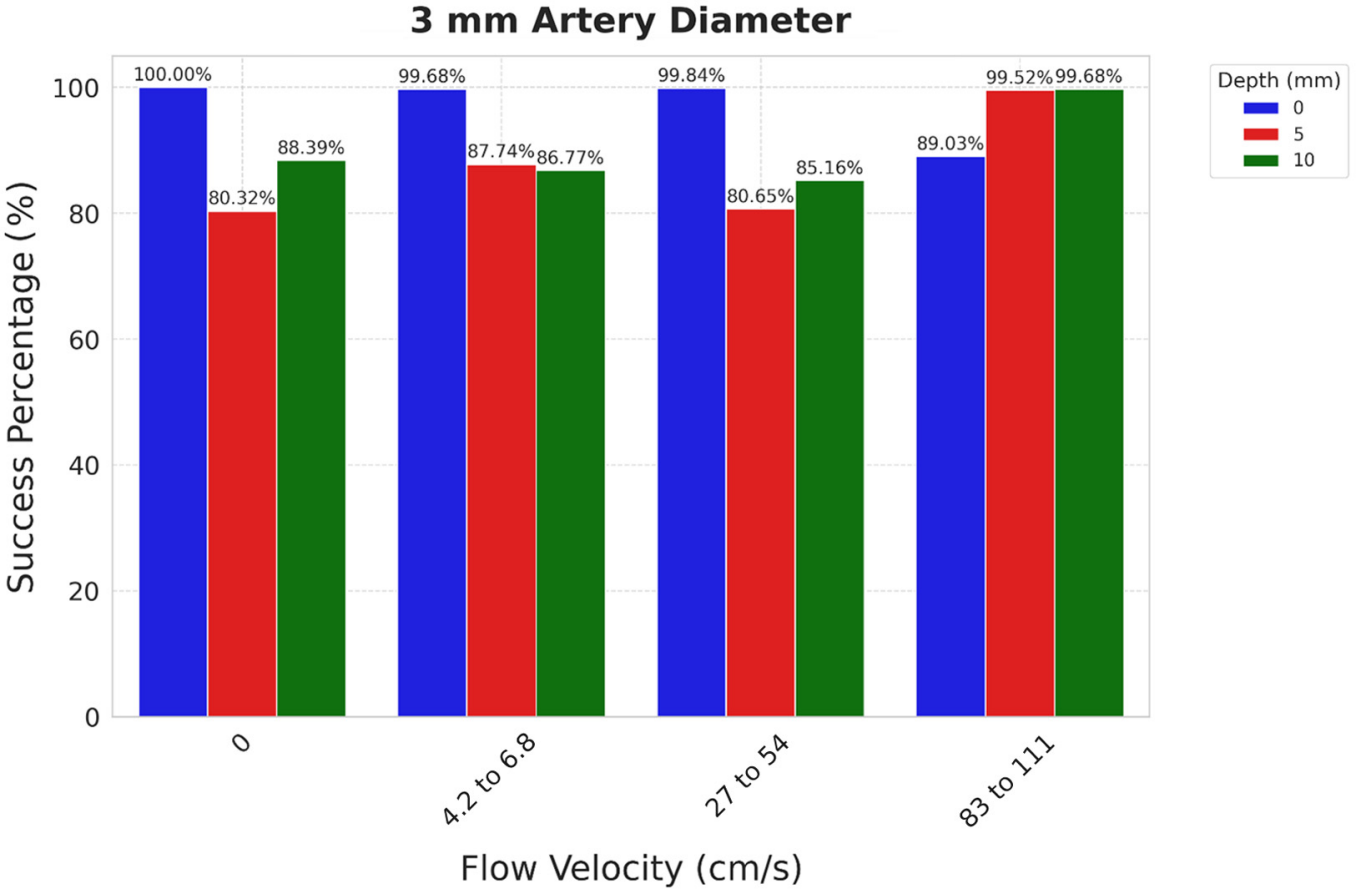
Flow velocity classification accuracy for 3 mm arteries. Accuracy in distinguishing zero, low, medium, and high flow at 0, 5, and 10 mm depths.

As the vessel depth increased from 0 to 5 and 10 mm, the success rates of the flow velocity detection varied between 80% and 89%, still significant for the flow evaluation (see Fig. 4). This reduction in accuracy can be attributed to the increased tissue interference, which attenuates the speckle patterns and makes it harder to detect flow conditions accurately. Despite this, the model maintained reasonable accuracy for low flow velocity, indicating that the pre-stroke conditions could also be detected.

**Fig. 5:**
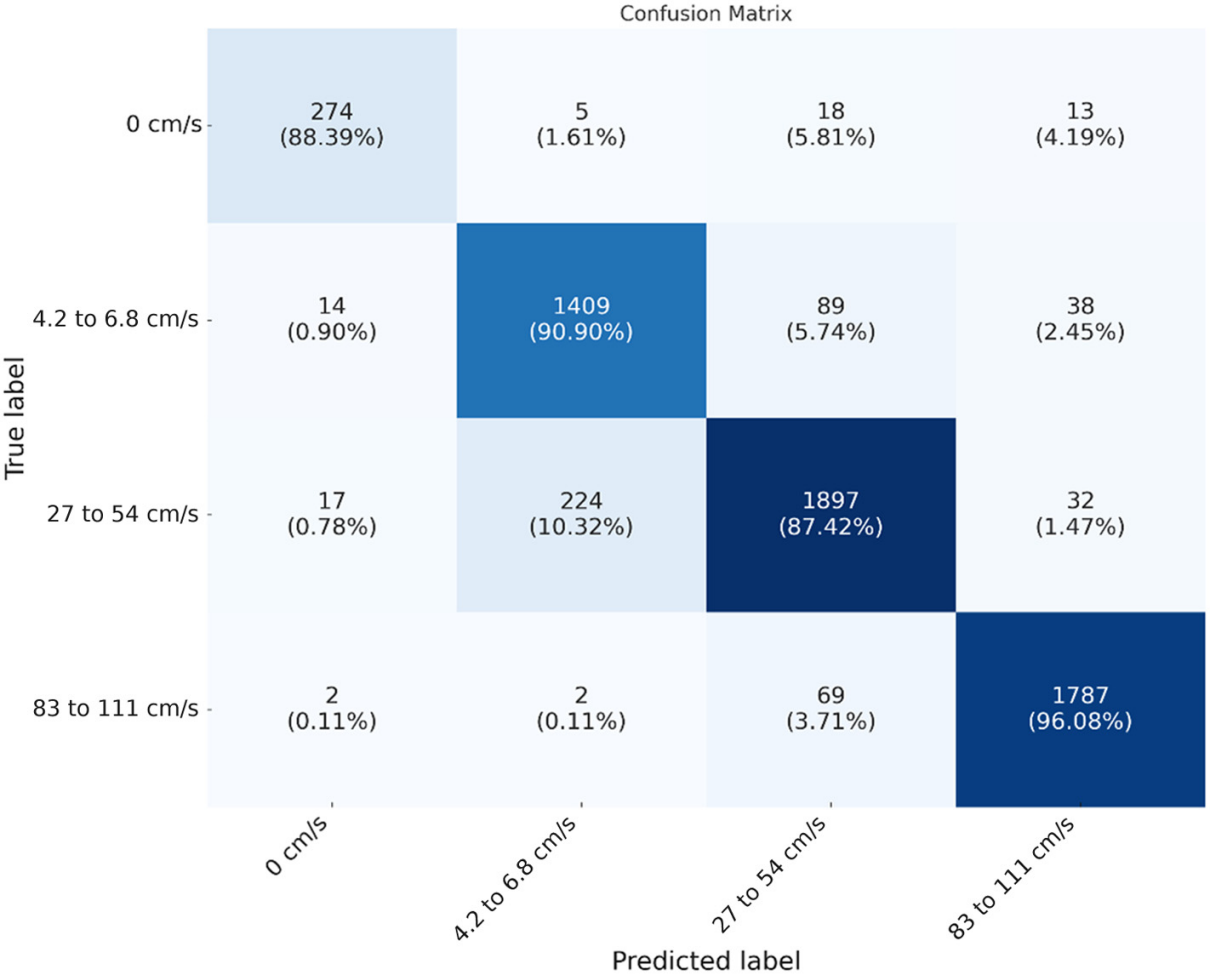
Confusion matrix for 3 mm artery flow classification. Diagonal cells show correct classifications; off-diagonal cells indicate misclassifications.

The confusion matrix for the 3 mm arteries at various depths (Fig. 5) provides a detailed view of the classification performance, showing the distribution of true and predicted labels.

The confusion matrix shows that the model accurately distinguishes among different flow conditions with minimal misclassifications. The highest misclassification rate is observed at 5 mm depth, where the system sometimes confuses low flow with medium flow. This highlights the challenge of detecting subtle differences in flow conditions through a deeper vessel. However, the classification rate at the 5 mm depth is high, reaching 87%.

### 3.2 Speckle Pattern Analysis for 6 mm Diameter Arteries

Flow velocities for 6 mm diameter vessels, simulating carotid arteries, were tested for 0 and 5 mm installation depth under the skin. We simulated a complete arterial blockage, adjusted the low rate in the range of 3 to 4 cm/s to simulate a pre-stroke condition, reduced flow velocity to 21 to 39 cm/s, and high flow in the 61 to 78 cm/s range.

**Fig. 6:**
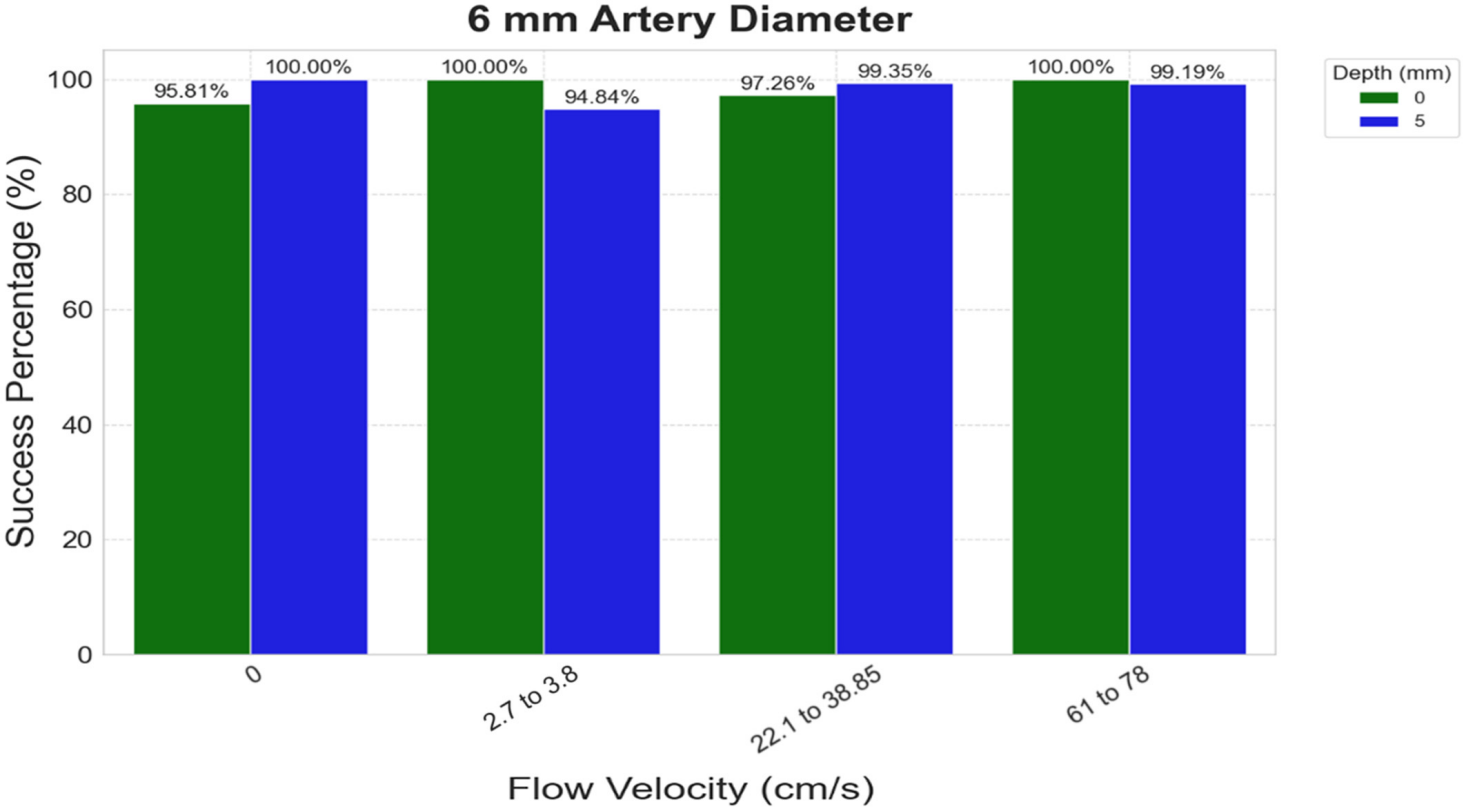
Flow velocity classification accuracy for 6 mm arteries. Four flow states (zero, low, medium, and high) were examined at depths of 0 and 5 mm, demonstrating generally higher accuracy compared with 3 mm vessels.

Figure 6 shows the success rates for detecting all mentioned flow conditions at 0 and 5 mm depth, which ranges from 95% to 100%.

This indicates that flow velocities of larger diameter arteries imbedded to 5 mm depth (similar to the carotid arteries) produce more robust speckle patterns, facilitating easier detection of flow changes, including partial or complete vessel blockage common for an ischemic stroke.

The confusion matrix for the 6 mm arteries provides a detailed view of the classification performance, showing the distribution of true and predicted labels.

**Fig. 7:**
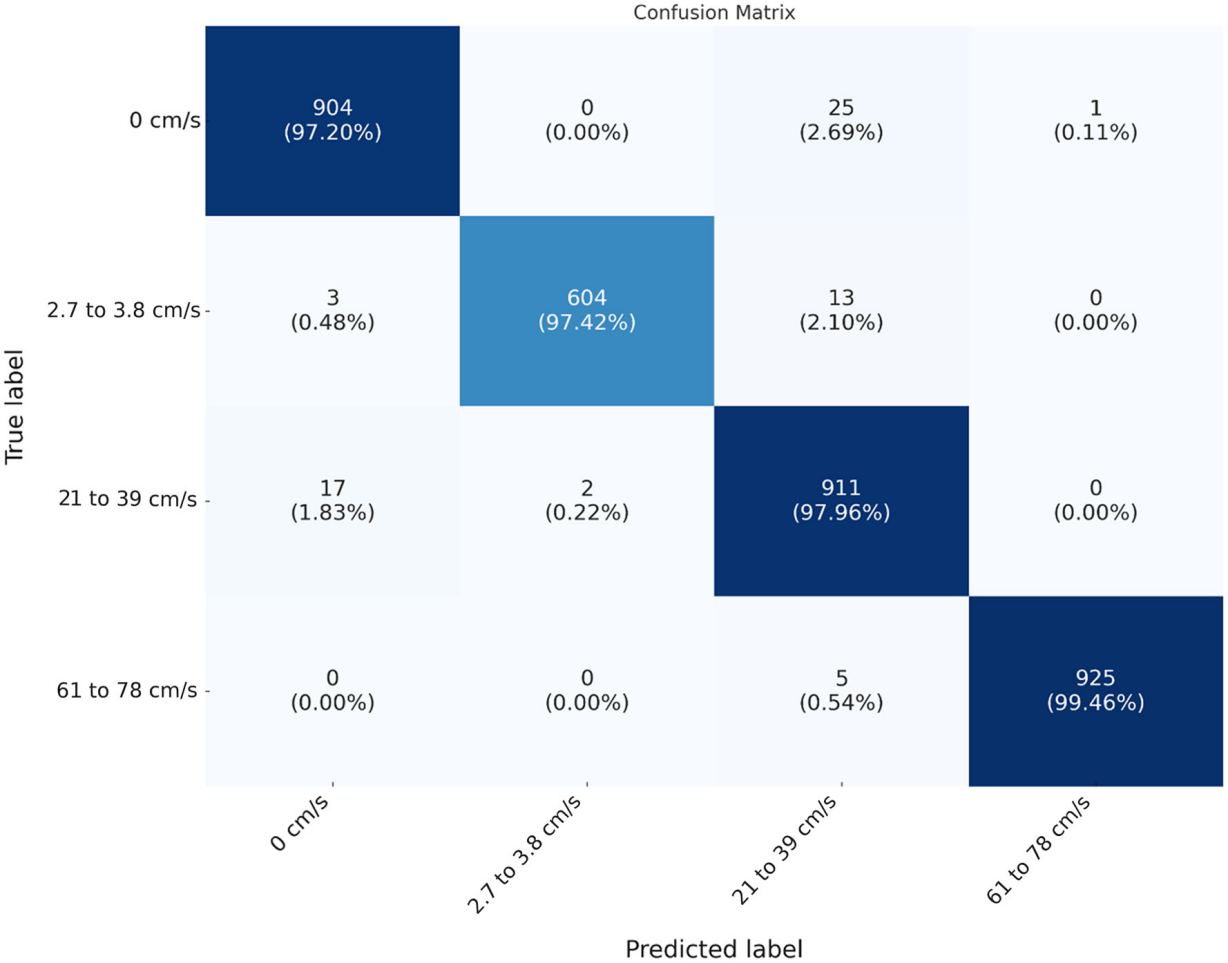
Confusion matrix for 6 mm artery flow classification. Minimal misclassifications underscore robust detection of zero, restricted, medium, and high flow states.

The confusion matrix (Fig. 7) shows that the model accurately distinguishes among different flow conditions for 6 mm arteries with minimal misclassifications. The highest misclassification rate is observed for no-flow conditions, where the system sometimes confuses no flow with medium flow, highlighting the challenge of precisely detecting subtle differences in flow conditions through a deeper blood vessel.

### 3.3 Comparative Analysis across Different Depths

A comparative analysis across different depths reveals that although deeper arteries produce attenuated speckle patterns, the system can still effectively detect flow changes. Performance metrics show that smaller-diameter arteries (3 mm) have slightly lower success rates than larger ones (6 mm). This difference is primarily due to the influence of the pulse generator. Smaller diameter arteries are more susceptible to fluctuations caused by the pulse generator, leading to greater variability in speckle patterns and slightly lower classification accuracy.

Interestingly, for high-velocity flow conditions, the classification performance at 0 mm depth under the skin can be lower than at in-deep positioning. One possible reason is that in surface vessel positioning, strong reflections and edge effects can lead to partial saturation or reduced contrast in the speckle signals, thereby degrading the deep learning model’s ability to discriminate flow states.

For other flow velocities, the results indicate that 5 mm depth often yields worse performance than 10 mm depth. In these intermediate layers, light scattering and reflections from multiple tissue boundaries can create more complex speckle patterns that partially obscure flow-induced fluctuations. Paradoxically, at 10 mm depth, although the overall signal is further attenuated, the speckle fluctuations may be relatively more stable and thus more readily distinguishable by the classifier.

The complete quantitative results for all tested flow velocities and artery diameters are summarized in Table 2.

**Table 2:** Classification performance for various artery diameters and flow velocity ranges, showing accuracy, precision, recall, and F1 score.

Artery diameter (mm)	Flow velocity (cm/s)	Number of test samples	Accuracy (%)	Precision	Recall	F1
3	0	310	88.39	0.89	0.88	0.88
3	4.2 to 6.8	1550	90.9	0.85	0.90	0.88
3	27 to 54	2170	87.4	0.91	0.87	0.89
3	83 to 111	1860	96	0.95	0.96	0.95
6	0	930	97.2	0.97	0.97	0.97
6	2.7 to 3.8	620	97.4	0.99	0.97	0.97
6	21 to 39	930	97.9	0.95	0.98	0.96
6	61 to 78	930	99.4	0.99	0.99	0.99

### 3.4 Deep Learning Model Performance

The X3D_M deep learning model was trained to classify flow states based on speckle pattern videos. The confusion matrices in Figs. 5 and 7 illustrate the distribution of true and predicted labels for 3 and 6 mm arteries, respectively. The model achieved high accuracy, distinguishing among different flow conditions with minimal misclassifications.

The detailed analysis and high performance of the X3D_M model underscore its potential for reliable, real-time stroke detection applications. The system’s robust capability across different experimental conditions suggests that it is a practical solution for early intervention and improved patient outcomes.

### 3.5 Comparison of LASCA Estimates under Defocused Geometry

To benchmark the proposed deep learning approach against a more traditional speckle technique, we applied a single‐point calibration–based LASCA to the same dataset. In LASCA, mean speckle contrast (K) in a reference video with known velocity is used to compute a proportionality constant A ($V\propto \frac{1}{K^{2}}$). Subsequent test videos are then processed under the assumption that the same relationship between contrast and velocity holds true. Although this method gave acceptable velocity estimates (within ∼10% to 20%) for videos recorded at the same depth and the exact same velocity as the calibration, a dramatic increase in error emerged when applying the same calibration across different depths.

For example, after calibrating on a medium‐flow vessel at 10 mm depth, LASCA‐estimated velocities at 5 mm depth overshot the true values by as much as 50% to 70%. In one instance, the actual flow speed of ∼28  cm/s was evaluated at ∼50  cm/s by LASCA, a mismatch of nearly 70%. Such discrepancies arise from LASCA’s in‐focus assumption and the requirement that scattering path lengths remain consistent. By contrast, our setup deliberately employs a defocused illumination to capture secondary speckle fields emanating from deeper tissue layers. These secondary speckles undergo more complex scattering, causing the simple ($V\propto \frac{1}{K^{2}}$). relationship to break down whenever the imaging depth or optical path length changes. Consequently, a single calibration constant cannot be expected to hold across multiple depths.

In practice, more elaborate calibration schemes (e.g., multipoint references) might reduce these LASCA errors, but the inherent sensitivity to depth and scattering conditions remains. Meanwhile, the deep learning–based strategy presented in this work sidesteps the reliance on a rigid contrast‐flow formula. By processing raw defocused speckle videos, it robustly adapts to multiple vessel depths and flow states without recalibration. This methodological difference clarifies why LASCA can face significant challenges when quantifying flow in defocused, multidepth configurations, whereas a data‐driven approach can accurately classify velocities under varying optical geometries.

### 3.6 Additional Experiments and Findings

We combined the zero and low-flow conditions to further improve the results. This approach aimed to simplify the classification problem by reducing the number of distinct flow states. However, this adjustment only yielded a marginal improvement in accuracy (∼1% to 2%). Given the minimal benefit, we retained the original four-label classification scheme. This decision was based on accurately distinguishing the different flow velocities, particularly those within the range observed in cerebral arteries.

## 4 Discussion

Our study demonstrates the potential of deep learning models, specifically the X3D_M architecture, for cost-effective remote stroke detection using laser speckle imaging. This quantitative discussion highlights the key metrics and performance variations observed across different artery diameters and depths, providing insights into the system’s strengths and limitations.

The results of our experiments show high success rates in detecting flow velocities across both 3 and 6 mm arteries. For 3 mm diameter arteries, the success rates ranged from 80% to 100% depending on the depth of the arteries and the flow conditions. At 0 mm depth, the model achieved a near-perfect detection rate of 100% for zero, low, and medium flows. However, as the vessel depth increased to 5 and 10 mm, the success rates dropped to around 85%. The attenuation of the speckle patterns due to increased tissue interference is likely responsible for this decline in performance. The confusion matrix for the 5 mm depth indicated occasional misclassifications between low and medium flows, reflecting the challenge of detecting subtle differences through deeper tissue. It is important to note that although the 3 mm arteries were chosen to approximate intracranial vessels such as the MCA, the phantom does not include a skull-like layer, so these results should be interpreted with caution regarding direct intracranial applicability.

For the 6 mm diameter arteries, which simulate larger vessels such as the carotid artery, the model demonstrated even higher success rates, ranging from 95% to 100%, across all depths (0 and 5 mm) and flow conditions. This superior performance can be attributed to the larger blood vessel diameter, which produces more distinct speckle patterns that are easier for the model to classify. The confusion matrix for the 6 mm arteries shows minimal misclassifications, with the highest confusion occurring between no-flow and medium-flow conditions. As the carotid artery is a superficial vessel, these results are more directly translatable to *in vivo* applications.

Although classification accuracy was slightly lower for the 3 mm artificial arteries than for the 6 mm ones, this finding remains clinically important because 3 mm approximates the diameter of MCA, one of the most frequently occluded vessels in ischemic stroke. Meanwhile, truly small penetrating arteries (∼1 to 2 mm), such as the lenticulostriates, are responsible for 20% to 25% of lacunar strokes,^43^ whereas medium-to-large cerebral vessels (2 to 6 mm) account for 30% to 50% of more severe events.^44^^,^^45^ Our system’s ability to classify zero-flow (ischemic) and partial-flow (pre-stroke) states, even at deeper tissue layers, demonstrates considerable promise for early detection across a range of clinically significant artery sizes. Future refinements could further enhance performance for smaller-caliber vessels, broadening the utility of this speckle-based approach in stroke diagnostics.

To simplify the classification problem, we combined the zero and low-flow conditions, reducing the number of distinct flow states. This modification slightly improved accuracy, with a 1% to 2% increase in the overall classification performance. However, given the minimal improvement, we retained the original four-label classification scheme to ensure the model accurately distinguishes among all flow velocities, particularly those within the range observed in cerebral arteries.

The results demonstrate that the X3D_M model effectively captures and classifies speckle pattern variations indicative of different blood flow conditions. The high success rates across multiple experimental conditions suggest that this approach is viable for real-time stroke detection, even in resource-limited settings.

A critical next step is extending this proof-of-concept to human cerebral vessels, where skull thickness, scalp layers, and patient movement introduce significant complexity. It must be emphasized that the current phantom lacks a skull-like layer, so although the data for 3 mm vessels (approximating intracranial arteries) are promising, they require further validation under realistic *in vivo* conditions. Several practical routes exist for achieving this goal. First, targeting “optical windows” such as the temporal region, where the skull is relatively thinner, can facilitate laser penetration, particularly in the NIR range, and allow speckle signals to be recorded from arteries such as the middle cerebral artery. For low-cost localization of the temporal window, standard scalp landmarks (as employed in transcranial Doppler exams) can be used to position the laser and camera without reliance on expensive imaging methods. Another option is to measure blood-flow changes in the neck’s carotid artery, which is more superficial and larger in diameter; clinically significant flow reductions detected there often precede ischemic events in the brain. Manual palpation of the carotid pulse can reliably guide sensor placement over the vessel, again avoiding the need for costly localization hardware. Enhanced detection strategies will be necessary, including high-sensitivity camera hardware and time-gated imaging to overcome strong scattering and absorption. Motion-compensation algorithms or short-time segment analyses will also help separate genuine flow-induced speckle fluctuations from patient movement artifacts. Progressively validating these methods *in vivo* beginning with animal models to refine hardware and imaging parameters and then proceeding to small-scale human pilot studies will clarify the practical limits for safe laser power, camera configurations, and AI algorithms. Although our next *in vivo* experiments will target cerebral arteries, the more immediately translatable carotid artery also remains a valuable clinical target.

Through these complementary efforts, the proposed approach can evolve into a viable noninvasive stroke screening tool.

## 5 Conclusion

This study introduces a rapid, cost-effective method for remote stroke detection using laser speckle imaging and deep learning models. Our findings confirm the method’s ability to accurately capture and classify blood flow changes, presenting a practical and accessible alternative to traditional imaging techniques such as CT and MRI. The X3D_M model’s impressive performance in processing speckle pattern videos underscores its potential for deployment in diverse settings, particularly in areas with limited resources.

Given that our current phantom system does not incorporate a skull-like layer, these results primarily support the feasibility of measuring superficial flow changes such as in the carotid artery where scattering is less severe than in true intracranial pathways. Although intracranial measurements remain a compelling longer-term objective, additional challenges (e.g., skull scattering, absorption, and the potential need for near-infrared illumination) must be addressed before translating this approach to cerebral arteries. Thus, our findings should be viewed as a proof of concept that lays the groundwork for further *in vivo* studies. Nevertheless, the proposed method promises to enhance stroke diagnostics by providing a rapid, noninvasive, and affordable solution. By facilitating early detection, this technique can potentially reduce neurological damage and improve recovery outcomes for stroke patients. The success of various labeling strategies, including depth-specific classifications, further validates the robustness of our approach. Our approach represents an important step toward making advanced stroke diagnostics more accessible and effective, particularly as we advance from superficial (e.g., carotid) to more challenging intracranial applications.

In conclusion, this study lays the groundwork for future research and development to optimize remote stroke detection technologies. Further developing, testing *in vivo*, and integrating this method into clinical practice could bridge the critical time gap before hospital-based confirmation with advanced imaging, thereby improving stroke treatment outcomes. Our approach represents a significant step in making advanced stroke diagnostics more accessible and effective, ultimately contributing to better patient care and quality of life.

## Data Availability

The code and data supporting the findings of this study are available from the corresponding author upon reasonable request.
